# Resmetirom attenuates atherosclerosis-associated dyslipidemia and vascular inflammation in high-fat diet-fed ApoE^−^/^−^ mice

**DOI:** 10.1371/journal.pone.0359161

**Published:** 2026-09-24

**Authors:** Xuedong Bai, Wenjie Fei, Jingzhou Fang, Chaomin Kong, Yaqi Xiang, Yuele Tian, Limin Wei

**Affiliations:** 1 Department of Internal Medicine, Hebei Medical University, Shijiazhuang, Hebei, China; 2 Department of Geriatric Cardiology, Hebei General Hospital, Shijiazhuang, Hebei, China; 3 Department of Endocrinology, Hebei General Hospital, Shijiazhuang, Hebei, China; 4 Department of Endocrinology, Langfang People’s Hospital, Langfang, Hebei, China; Charite Universitatsmedizin Berlin, GERMANY

## Abstract

**Background:**

Atherosclerosis is driven by interacting lipid and inflammatory pathways. There remains a pressing clinical need for drugs that exert both lipid-lowering and vascular anti-inflammatory effects. Studies have suggested that thyroid hormone receptor β (THR-β) agonists can alleviate atherosclerosis while avoiding the cardiotoxic adverse effects associated with thyroid hormone. Resmetirom is an oral, once-daily, liver-targeted selective THR-β agonist. It has attracted attention for improving lipid metabolism, yet its effects on vascular inflammatory phenotypes remain poorly clarified. We investigated the effects of resmetirom on atherosclerosis-associated dyslipidemia, plaque burden and vascular inflammatory phenotypes.

**Methods:**

Apolipoprotein E-deficient (ApoE^-^/^-^) mice fed a high-fat diet (HFD) for 8 weeks were randomized to vehicle, low-dose (3 mg/kg/day), or high-dose (10 mg/kg/day) resmetirom for eight weeks (*n* = 10 per group). Serum lipids, inflammatory cytokines, aortic plaque morphology, NLRP3 inflammasome signaling, NF-κB activation, oxidative stress, and macrophage polarization were analyzed by one-way ANOVA with Tukey post hoc testing; dose-response regression was performed across disease-bearing groups.

**Results:**

Resmetirom treatment reduced aortic plaque burden, corrected atherogenic dyslipidemia, and suppressed aortic NLRP3 and caspase-1 expression in a dose-dependent manner. High-dose resmetirom lowered low-density lipoprotein cholesterol (LDL-C) by 39.48%, and reduced aortic lipid accumulation on Oil Red O staining compared to model controls. Additionally, it suppressed interleukin-1β (IL-1β) by 59.28%; Western blotting showed lower p-p65/total p65 ratio and higher IκBα/GAPDH abundance. Flow-cytometric ROS intensity decreased by 35.56%, and the M1/M2 macrophage ratio decreased by 72.81%. Exploratory cross-assay analyses showed coherent associations among systemic cytokines and aortic NF-κB/ROS/macrophage readouts.

**Conclusions:**

Resmetirom attenuated atherosclerosis-associated dyslipidemia, plaque burden and vascular inflammation in HFD ApoE^−/−^ mice. The data support an association between resmetirom treatment and suppression of the NF-κB/ROS-NLRP3 inflammatory phenotype, warranting mechanistic validation in models with direct pathway perturbation. These findings support its translational potential in atherosclerotic cardiovascular disease.

## Introduction

Atherosclerosis is driven by interacting lipid and inflammatory pathways [1,2]. Lipoprotein retention, endothelial activation and maladaptive immune responses promote lesion formation [3]. Despite advances in lipid-lowering therapies, substantial residual cardiovascular risk persists, and inflammatory pathways that drive plaque progression independently of cholesterol levels remain an important therapeutic target [4,5].

Clinical evidence that interleukin-1β (IL-1β) inhibition reduces recurrent cardiovascular events without directly lowering lipids supports vascular inflammation as a treatment-relevant axis [6,7]. Mechanistically, cholesterol crystals and other danger signals activate the NOD-like receptor family pyrin domain-containing 3 (NLRP3) inflammasome and IL-1 pathway, and this response is amplified by NF-κB priming, oxidative stress and macrophage inflammatory polarization [8–11].

Thyroid hormone receptor β (THR-β) signaling regulates hepatic lipid handling, cholesterol metabolism and lipoprotein clearance [12,13]. Beyond its metabolic effects, emerging evidence suggests that THR-β signaling modulates inflammatory responses [14,15]. Resmetirom (MGL-3196) is a first-in-class, liver-directed THR-β agonist that improves metabolic and histological endpoints in non-alcoholic steatohepatitis (NASH)/metabolic dysfunction-associated steatohepatitis (MASH) studies [16–18]. However, whether resmetirom exerts direct anti-inflammatory actions relevant to atherosclerosis remains unexplored.

Because dyslipidemia, oxidative stress and inflammasome-related inflammation overlap in metabolic liver disease and atherosclerosis [19], we tested whether resmetirom attenuates lipid, plaque and vascular inflammatory readouts in high-fat diet (HFD)-fed apolipoprotein E-deficient (ApoE^-^/^-^) mice, a well-validated model of accelerated atherosclerosis [20]. We hypothesized that resmetirom would reduce atherosclerosis-associated dyslipidemia and plaque burden and that these effects would be accompanied by suppression of NF-κB/ROS-NLRP3 inflammatory markers.

## Materials and methods

### Animals diet and treatment

Male C57BL/6J WT (wild-type) and ApoE-/- mice (8 weeks old, Beijing Vital River, Beijing, China) were housed under specific-pathogen-free (SPF) conditions with ad libitum access to food and water. Forty-five mice were used: 10 WT (normal control, NC) fed standard chow, and 35 ApoE-/- mice fed a HFD (21% fat, 0.15% cholesterol; D12079B, Research Diets, New Brunswick, NJ, USA) for 8 weeks. Five ApoE-/- animals were euthanized after 8 weeks of HFD feeding to verify successful atherosclerosis induction and were not assigned to endpoint analyses. After confirming model establishment, the remaining 30 ApoE-/- mice were randomized by computer-generated numbers to model control (MC, vehicle), low-dose resmetirom (LD, 3 mg/kg/day) or high-dose resmetirom (HD, 10 mg/kg/day) groups (n = 10/group). Resmetirom (MGL-3196; MedChemExpress, Monmouth Junction, NJ, USA) was suspended in 0.5% carboxymethylcellulose and administered once daily by oral gavage for 8 weeks during continued HFD; MC mice received equal-volume vehicle. The 3 mg/kg/day dose was selected as a literature-supported pharmacologically active dose, whereas 10 mg/kg/day was included as a higher exposure dose to assess dose responsiveness [21,22]. Published pharmacokinetic data indicate that 3 mg/kg in mice provides exposure of the same order as clinically used resmetirom regimens, whereas 10 mg/kg represents a supra-clinical exposure extension [22,23]. After treatment, mice were fasted for 12 h and anesthetized with intraperitoneal injection of sodium pentobarbital (P8830, Solarbio, Beijing, China) at a dose of 55 mg/kg with 1% concentration. Blood was harvested via cardiac puncture, and animals were euthanized by exsanguination and aortic tissue harvest under deep anesthesia. Tissue collection, IHC scoring, Western blot quantification, RT-qPCR and flow-cytometric gating were performed blinded to group assignment.

All procedures were approved by the Ethics Committee of Hebei General Hospital (approval No. 202385). The study was reported in accordance with the ARRIVE 2.0 guidelines [24].

### Serum lipid and cytokine measurements

After treatment, mice were fasted for 12 h and anesthetized with sodium pentobarbital at a dose of 55 mg/kg (1% solution). Blood was collected by cardiac puncture, clotted and centrifuged at 3,000 × g for 10 min; serum was stored at −80 °C. Total cholesterol (TC), triglycerides (TG), HDL-cholesterol (HDL-C) and LDL-cholesterol (LDL-C) were measured enzymatically. IL-1β, IL-18, TNF-α and C-reactive protein (CRP) were measured by ELISA.

### Aortic histology and plaque quantification

Aortas were perfused with ice-cold phosphate-buffered saline and collected for histology, Oil Red O morphometry and molecular assays. Aortic-root sections were stained with hematoxylin-eosin for morphology and Oil Red O for lipid-rich lesions. Images were analyzed with ImageJ/Fiji [25,26]. Three sections per animal were averaged to one biological replicate. Morphometric endpoints were aortic plaque area, Oil Red O-positive area, Oil Red O-positive fraction and intima-media ratio.

### Immunohistochemistry

Paraffin sections were deparaffinized, antigen-retrieved, blocked and incubated overnight with antibodies against caspase-1, IL-1β, MCP-1 and NLRP3. HRP-conjugated secondary antibodies and 3,3’-diaminobenzidine were used for detection, followed by hematoxylin counterstaining. Integrated optical density was measured in three non-overlapping fields per section and averaged to one biological value per animal. Primary-antibody suppliers, catalog numbers and working dilutions are provided in S1 Table.

### Western blotting

Aortic lysates were separated by SDS-PAGE and transferred to PVDF membranes. Membranes were probed for NLRP3, caspase-1, IL-1β, MCP-1, phospho-p65 (Ser536), total p65, IκBα and GAPDH. Band intensities were quantified by densitometry using ImageJ/Fiji. NLRP3, caspase-1, IL-1β, MCP-1 and IκBα were normalized to GAPDH; phospho-p65 was normalized to total p65 and analyzed as the p-p65/total p65 ratio. Western blot antibody details are provided in S1 Table.

### RT-qPCR

Total aortic RNA was reverse-transcribed to cDNA. Real-time PCR quantified Nlrp3, Casp1, Il1b and Ccl2, with Actb as the reference gene. Relative expression was calculated by the 2^ − ΔΔCt method using the NC mean as baseline.

### Flow cytometry

Aortic tissue was enzymatically dissociated and filtered to single-cell suspensions. Macrophages were gated as CD45 + F4/80 + CD11b+ events; M1 and M2 subsets were defined by CD86 and CD206 positivity, respectively. Intracellular ROS was measured by CellROX median fluorescence intensity, and the M1/M2 ratio was calculated per animal. Flow-cytometry antibody, clone, fluorochrome and amount details are provided in S1 Table.

### Statistical analysis

Continuous variables are reported as mean ± SD. Group comparisons were evaluated by one-way ANOVA with Tukey HSD post hoc testing. Ordinary least squares regression was used to assess dose-response relationships. Relative restoration toward NC was calculated for disease-increased endpoints. Correlation, principal component and mediation analyses were performed for exploratory purposes. Mediation analysis adopted the Baron-Kenny framework. All statistical analyses were performed using Python 3.11. Data processing and management were conducted with pandas (version 2.2.3). One-way analysis of variance (ANOVA) was performed using statsmodels (version 0.14.4), followed by Tukey’s honestly significant difference (HSD) post hoc test for pairwise comparisons. Data visualization was carried out with matplotlib (version 3.10.3). Results are expressed as mean ± SD, and statistical significance was set at p < 0.05.

## Results

### Resmetirom improves atherogenic dyslipidemia

**Fig 1:**
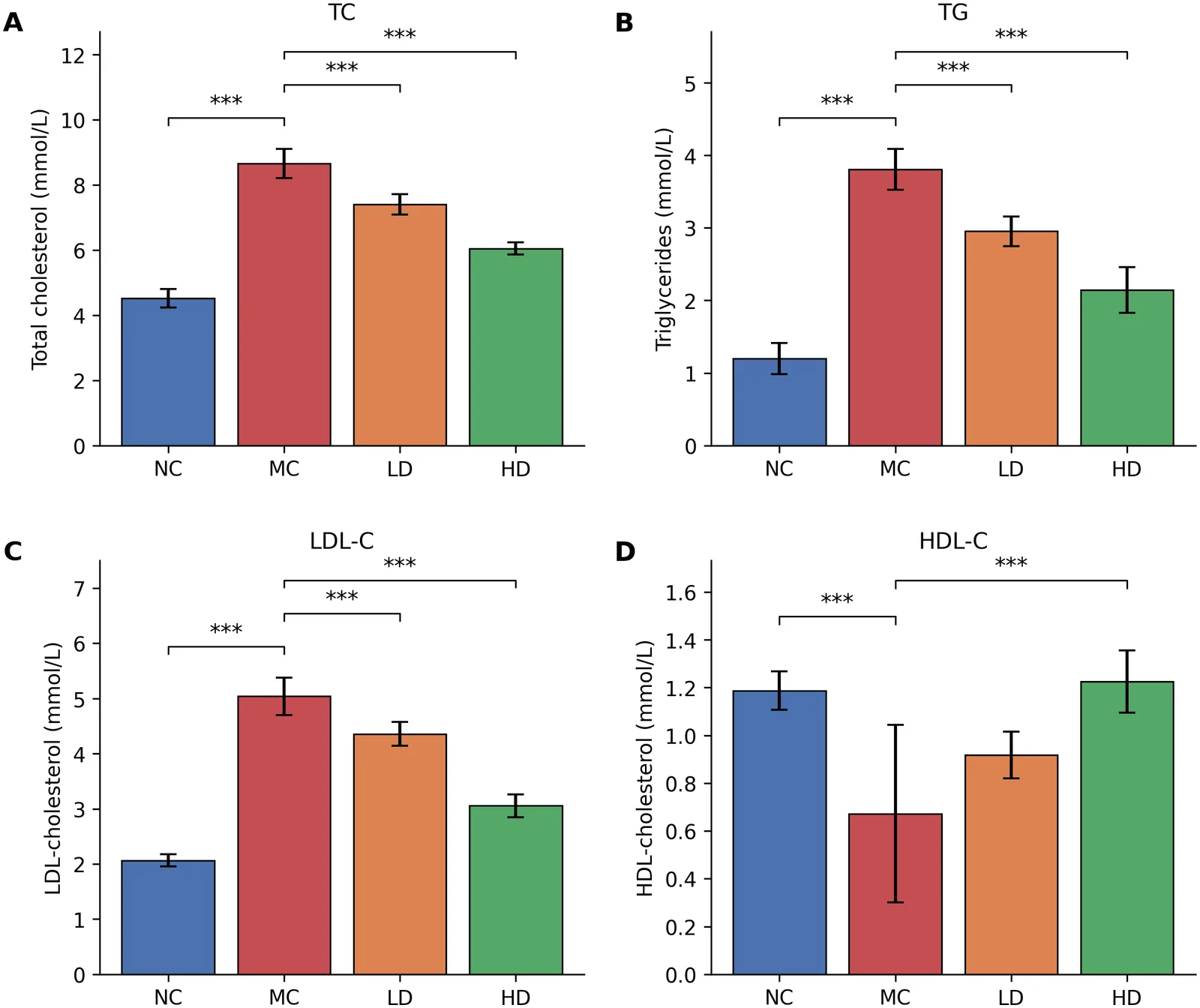
Dose-dependent improvement of atherogenic dyslipidemia by resmetirom in ApoE^−/−^ mice. (A) Serum total cholesterol. (B) Serum triglycerides. (C) LDL-cholesterol. (D) HDL-cholesterol. Bars are mean ± SD; n = 10/group. One-way ANOVA with Tukey HSD vs MC (*p < 0.05, p < 0.01***,** *p* < 0.001).

Resmetirom dose-dependently restored all four serum-lipid endpoints toward the normal-control profile. MC mice showed marked dyslipidemia compared with NC mice, including higher TC (8.66 ± 0.45 vs 4.52 ± 0.29 mmol/L), TG (3.81 ± 0.28 vs 1.20 ± 0.21 mmol/L) and LDL-C (5.04 ± 0.34 vs 2.06 ± 0.11 mmol/L), together with lower HDL-C (0.67 ± 0.37 vs 1.19 ± 0.08 mmol/L). High-dose resmetirom reduced TC to 6.05 ± 0.19 mmol/L, TG to 2.15 ± 0.32 mmol/L and LDL-C to 3.05 ± 0.20 mmol/L and increased HDL-C to 1.23 ± 0.13 mmol/L. Dose-response regression across MC, LD and HD was significant for TC (β = −0.25, R^2^ = 0.88), TG (β = −0.16, R^2^ = 0.82), LDL-C (β = −0.20, R^2^ = 0.92) and HDL-C (β = +0.05, R^2^ = 0.50; all *p* < 0.001). At the high dose, relative restoration was 63.2 ± 4.5% for TC, 63.8 ± 12.1% for TG, 66.7 ± 6.9% for LDL-C and 107.6 ± 25.4% for HDL-C (Fig 1).

### Resmetirom reduces aortic plaque burden

**Fig 2:**
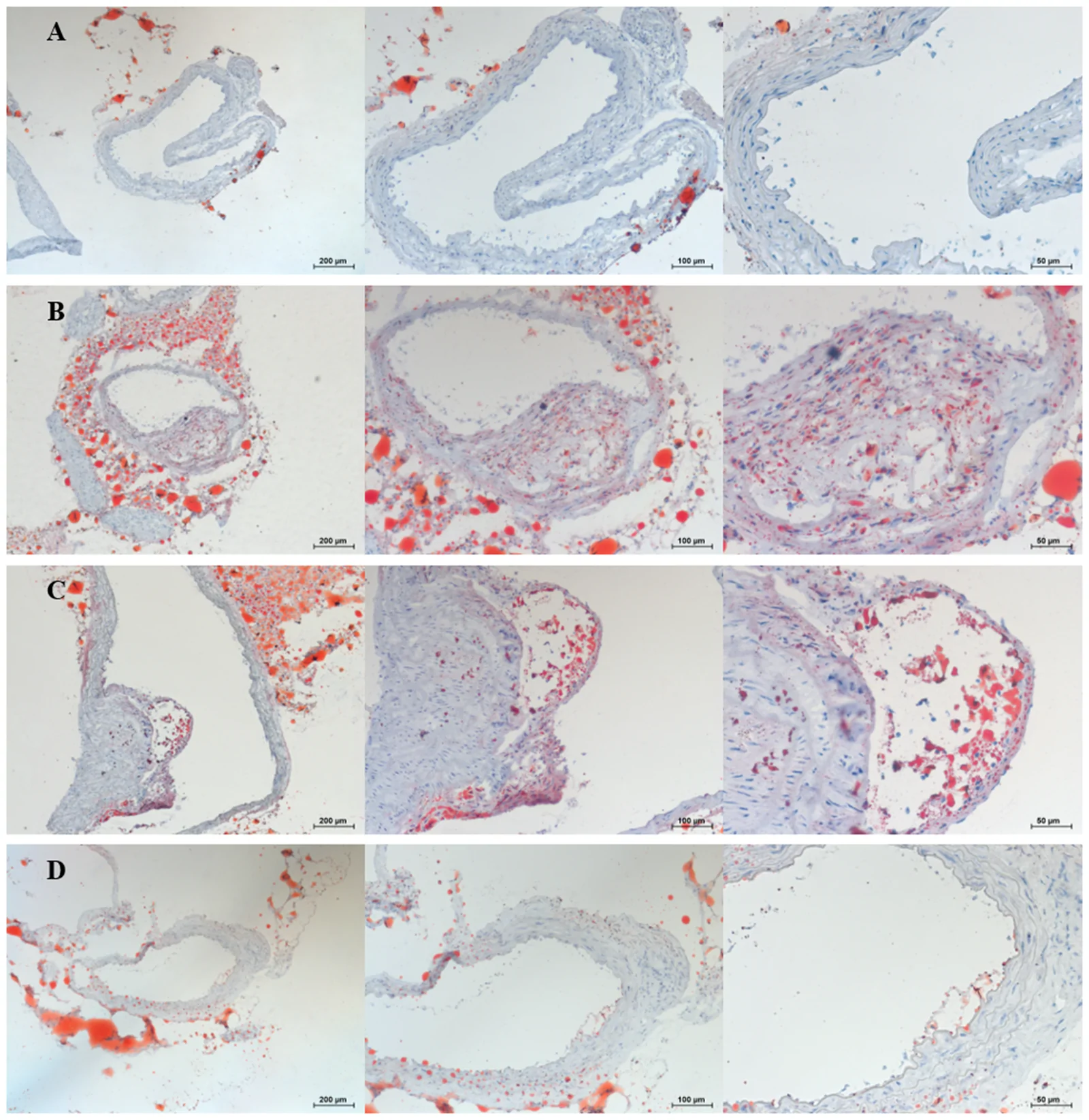
Representative aortic-root histology. Representative hematoxylin-eosin and Oil Red O staining of aortic-root sections from NC, MC, LD and HD groups. Scale bars are shown on the images.

**Fig 3:**
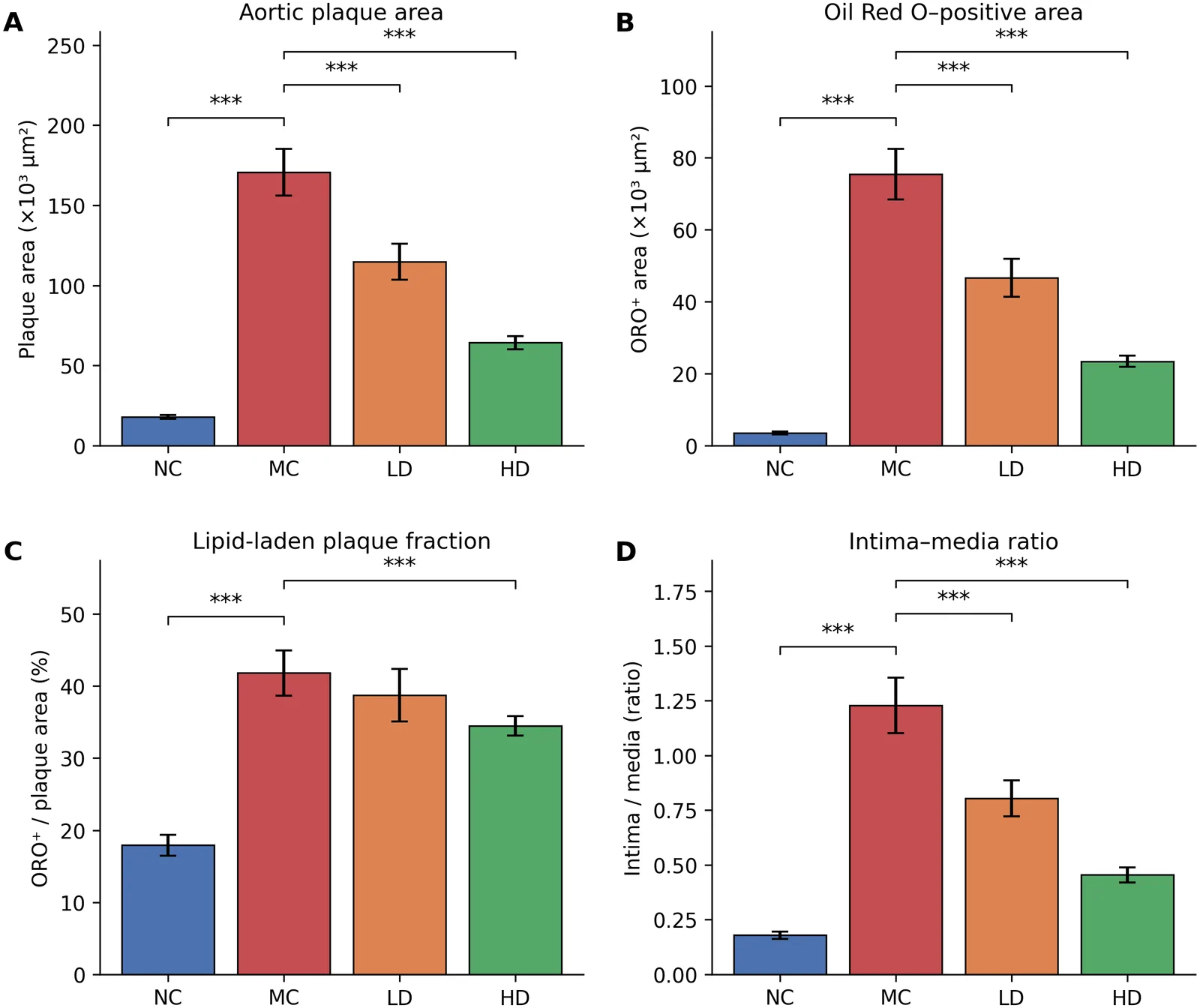
Quantitative aortic plaque morphometry on Oil Red O-stained aortic-root sections. (A) Aortic plaque area. (B) Oil Red O-positive area. (C) Oil Red O-positive fraction. (D) Intima-media ratio. Bars are mean ± SD; n = 10 animals/group after averaging three sections per animal.

Representative hematoxylin-eosin and Oil Red O images showed extensive aortic-root lesions in MC mice, with visibly smaller lesions in the LD and HD groups (Fig 2). Quantitative morphometry on Oil Red O-stained aortic-root sections confirmed a dose-related reduction in plaque burden (Figs 3 and S1). Plaque area increased from 17.85 ± 1.36 × 10^3^ μm^2^ in NC to 170.73 ± 14.64 × 10^3^ μm^2^ in MC and decreased to 114.74 ± 11.28 × 10^3^ μm^2^ with LD and 64.28 ± 4.07 × 10^3^ μm^2^ with HD (both *p* < 0.001 vs MC). Oil Red O-positive area followed a similar pattern (NC 3.51 ± 0.43, MC 75.46 ± 7.08, LD 46.66 ± 5.30, HD 23.43 ± 1.56 × 10^3^ μm^2^). The intima-media ratio decreased from 1.23 ± 0.13 in MC to 0.80 ± 0.08 in LD and 0.45 ± 0.03 in HD. The Oil Red O-positive fraction was 17.92 ± 1.43% in NC, 41.83 ± 3.15% in MC, 38.76 ± 3.65% in LD and 34.49 ± 1.37% in HD; compared with MC, the high-dose group showed a significant reduction (p < 0.001), whereas the low-dose change was borderline and did not reach significance (p = 0.058).

### Resmetirom suppresses systemic inflammatory cytokines

**Fig 4:**
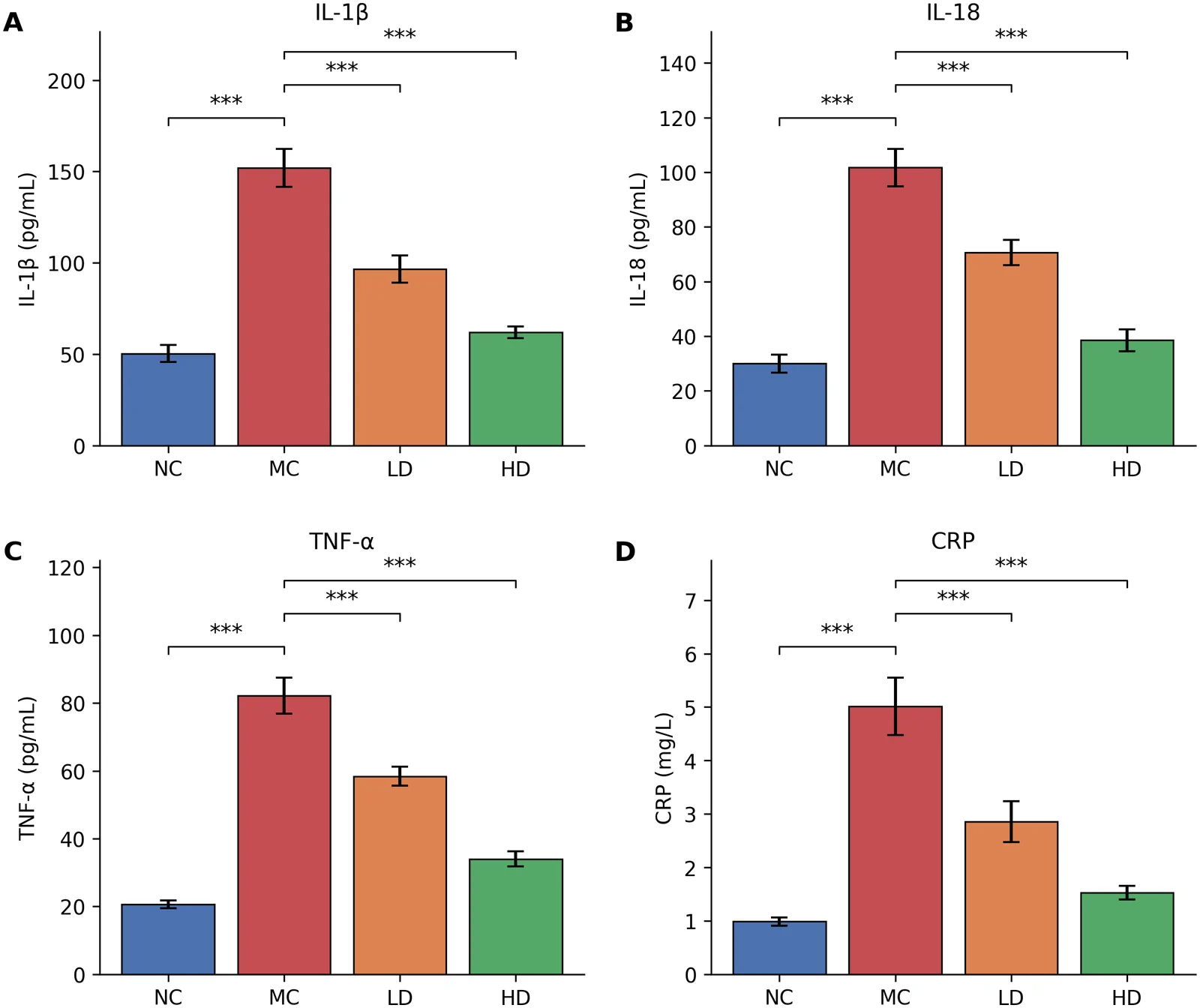
Serum inflammatory cytokine profile. (A) IL-1β. (B) IL-18. (C) TNF-α. (D) CRP. Bars are mean ± SD; n = 10/group. One-way ANOVA with Tukey HSD vs MC.

Resmetirom suppressed all four major pro-inflammatory cytokines in a dose-related manner (Fig 4). Serum IL-1β rose from 50.37 ± 4.77 pg/mL in NC to 152.09 ± 10.38 pg/mL in MC and decreased to 96.60 ± 7.45 pg/mL with LD and 61.93 ± 3.16 pg/mL with HD. IL-18 decreased from 101.77 ± 6.85 pg/mL in MC to 70.62 ± 4.68 pg/mL in LD and 38.55 ± 3.98 pg/mL in HD. TNF-α decreased from 82.19 ± 5.34 pg/mL in MC to 58.42 ± 2.82 pg/mL in LD and 34.03 ± 2.20 pg/mL in HD. CRP decreased from 5.01 ± 0.54 mg/L in MC to 2.86 ± 0.38 mg/L in LD and 1.53 ± 0.13 mg/L in HD. Dose-response slopes were significant for all four cytokines (all *p* < 0.001).

### Aortic inflammasome markers are reduced at IHC and transcript levels

**Fig 5:**
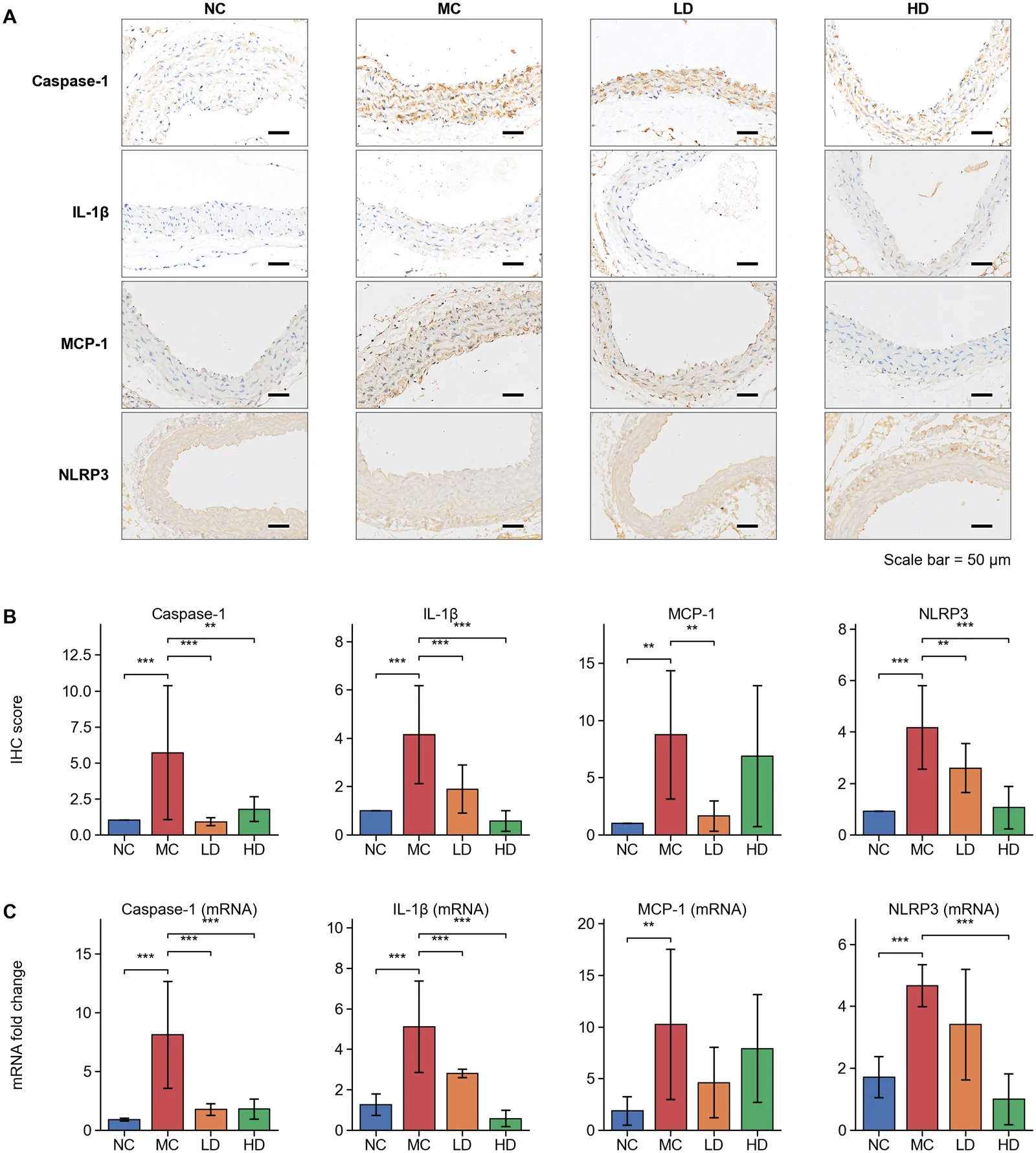
Representative IHC staining and quantification of aortic inflammatory markers. (A) Representative IHC staining for caspase-1, IL-1β, MCP-1 and NLRP3 in aortic-root sections from the NC, MC, LD and HD groups. Scale bars, 50 μm. (B) Corresponding IHC scores. (C) Relative mRNA expression of Casp1, Il1b, Ccl2 and Nlrp3 measured by RT-qPCR. Data are mean ± SD (n = 10/group). **p < 0.01 and ***p < 0.001 by one-way ANOVA followed by Tukey’s HSD test.

Aortic inflammatory markers were reduced overall by resmetirom, with IL-1β and NLRP3 showing the clearest dose-related pattern, whereas caspase-1 and MCP-1 showed less consistent responses at the tissue protein and mRNA levels (Figs 5 and S2). Caspase-1 was reduced at both treatment doses, but incremental suppression at HD was not evident (IHC 0.92 ± 0.29 at LD vs 1.79 ± 0.85 at HD; mRNA 1.76 ± 0.50 at LD vs 1.80 ± 0.84 at HD). MCP-1 was reduced at LD by IHC (1.65 ± 1.32; *p* < 0.01 vs MC) but showed greater variability at HD (6.89 ± 6.17; *p* = 0.760 vs MC).

### Western blotting confirms reduced inflammasome protein abundance and NF-κB activation

**Fig 6:**
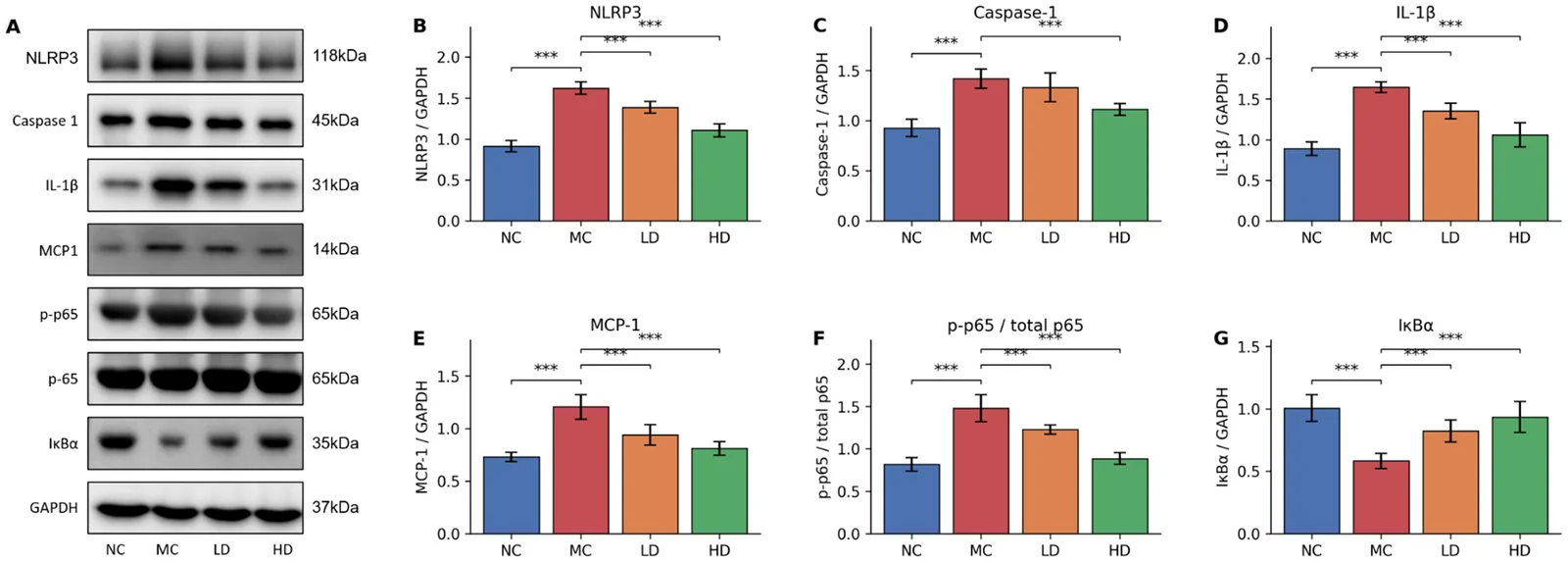
Western blot analysis of aortic inflammasome proteins and NF-κB activation. (A) Representative Western blots. (B) NLRP3/GAPDH. (C) Caspase-1/GAPDH. (D) IL-1β/GAPDH. (E) MCP-1/GAPDH. (F) p-p65/total p65. (G) IκBα/GAPDH. Bars are mean ± SD; n = 10/group. Band intensities were quantified by densitometry; p-p65 was normalised to total p65, while other proteins were normalised to GAPDH.

Western blotting confirmed reduced inflammasome protein abundance and NF-κB activation (Fig 6). In MC mice, NLRP3/GAPDH, caspase-1/GAPDH, IL-1β/GAPDH and MCP-1/GAPDH were increased to 1.62 ± 0.07, 1.42 ± 0.10, 1.64 ± 0.06 and 1.20 ± 0.12, respectively. At the high dose, these values decreased to 1.11 ± 0.08, 1.11 ± 0.06, 1.06 ± 0.15 and 0.81 ± 0.06. The p-p65/total p65 ratio decreased from 1.48 ± 0.16 in MC to 1.23 ± 0.06 in LD and 0.88 ± 0.07 in HD, whereas IκBα/GAPDH increased from 0.58 ± 0.06 in MC to 0.82 ± 0.09 in LD and 0.93 ± 0.12 in HD. High-dose restoration toward NC was 89.6 ± 10.3% for p-p65/total p65 and 83.1 ± 29.4% for IκBα.

### Resmetirom reduces aortic ROS and shifts macrophage polarization

**Fig 7:**
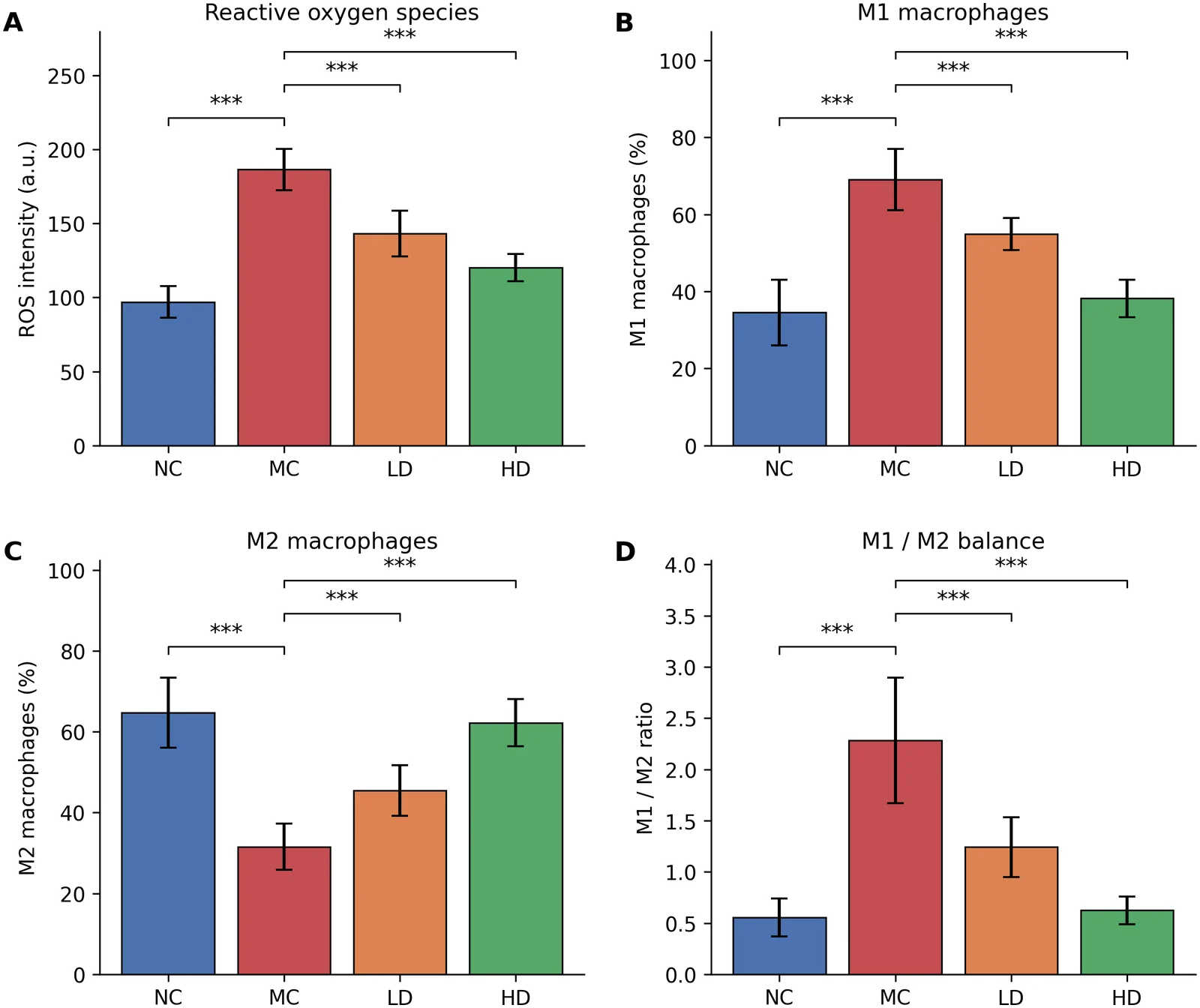
Aortic ROS and macrophage polarization by flow cytometry. (A) ROS intensity. (B) M1 macrophage frequency. (C) M2 macrophage frequency. (D) M1/M2 ratio. Bars are mean ± SD; n = 10/group.

Resmetirom reduced aortic ROS and shifted macrophage polarization toward a less pro-inflammatory profile by flow cytometry (Figs 7 and S3). ROS intensity increased from 96.91 ± 10.62 in NC to 186.36 ± 14.04 in MC and decreased to 143.00 ± 15.46 in LD and 120.09 ± 9.25 in HD. M1 macrophage frequency decreased from 69.07 ± 7.97% in MC to 54.92 ± 4.15% in LD and 38.18 ± 4.87% in HD. M2 macrophage frequency increased from 31.51 ± 5.72% in MC to 45.46 ± 6.30% in LD and 62.22 ± 5.84% in HD. The M1/M2 ratio decreased from 2.28 ± 0.61 in MC to 1.24 ± 0.29 in LD and 0.62 ± 0.13 in HD. Dose-response regression remained significant for ROS, M1%, M2% and M1/M2 ratio (all p < 0.001).

### Exploratory integrative analyses

Exploratory cross-assay correlation, PCA and mediation analyses showed internally coherent associations among systemic cytokines and aortic NF-κB/ROS/macrophage readouts (S4 and S5 Figs).

## Discussion

This study shows that resmetirom attenuated atherosclerosis-associated dyslipidemia, plaque burden and vascular inflammation in HFD-fed ApoE − / − mice, consistent with prior investigations on THR-β agonists GC-1 and KB3495 [27,28]. The strongest and most consistent findings were improvement of atherogenic lipid parameters, reduction of aortic plaque area and Oil Red O-positive lesion area, suppression of circulating IL-1β/IL-18/TNF-α/CRP, and lower aortic inflammasome/NF-κB/ROS/macrophage inflammatory readouts. The data fit current concepts that lipid metabolism and inflammation are interacting rather than isolated therapeutic domains in atherosclerosis [29,30]. These converging metabolic and anti-inflammatory mechanisms position resmetirom as a promising therapeutic repurposing candidate for atherosclerotic cardiovascular disease, particularly in patients with comorbid MASH who face elevated cardiovascular risk and would benefit from integrated hepato-vascular protection.

The lipid and plaque data provide the most direct evidence of efficacy. Improvement in TC, TG, LDL-C and HDL-C is compatible with THR-β-mediated regulation of hepatic lipid metabolism and lipoprotein handling [12,31]. Clinical trials of resmetirom in NASH/MASH also report improvements in liver fat and metabolic biomarkers [18,32]. Our data extend this metabolic rationale to an atherosclerosis-prone mouse model. The smaller change in Oil Red O-positive fraction suggests that lesion size improved more clearly than lesion composition; therefore, these histological findings support attenuation of plaque burden and provide a basis for future investigations into plaque stabilisation.

The inflammatory results are also consistent with the central role of IL-1 signaling and NLRP3 activation in vascular inflammation [7,33]. Resmetirom reduced circulating IL-1β, IL-18, TNF-α and CRP, lowered aortic IL-1β/NLRP3 readouts at protein and transcript levels, decreased the p-p65/total p65 ratio and restored IκBα abundance. This profile is consistent with lower activity of NF-κB- and inflammasome-related processes, both of which are central to cholesterol-crystal and IL-1/NLRP3 signalling in atherosclerosis [34]. These pathway-associated readouts support an associative interpretation, while a causal contribution to plaque and inflammatory changes requires confirmation through direct pathway perturbation. MCP-1 and caspase-1 showed less consistent dose-dependent patterns, possibly reflecting biological heterogeneity or variability in whole-vessel measurements. The accompanying reductions in ROS and M1/M2 ratio further support a less inflammatory aortic microenvironment. Our M1/M2 data should be interpreted as first-order indicators of polarization trends, as monocyte recruitment involves multiple chemokine pathways beyond the traditional M1/M2 framework [35]. Future single-cell or spatial profiling would be needed to define whether resmetirom changes specific lesional macrophage subpopulations.

The exploratory correlation, PCA and statistical mediation analyses demonstrated cross-assay coherence. In the absence of direct pathway perturbation, the mediation findings are best interpreted as hypothesis-generating evidence rather than causal mediation. This interpretation is consistent with current interest in targeted inflammatory management of atherosclerotic disease [2,36].

These findings have translational relevance because resmetirom has entered the clinical management landscape for eligible patients with non-cirrhotic MASH and significant fibrosis [21,32,37]. MASLD/MASH commonly coexists with cardiometabolic risk, reflecting shared hepatic and vascular metabolic-inflammatory pathways [37,38]. The present data identify a clinically testable question: whether patients receiving resmetirom show favourable changes in lipid profile, inflammatory biomarkers and vascular imaging endpoints. However, the direct effects of resmetirom on ASCVD outcomes remain to be established [38]. Such studies would align with broader efforts to address residual inflammatory risk in atherosclerotic cardiovascular disease.

## Conclusion

Resmetirom improved dyslipidemia, reduced aortic plaque burden and was associated with a less inflammatory vascular phenotype in HFD-fed ApoE^−/−^ mice. The findings support an association between resmetirom treatment and suppression of NF-κB/ROS-NLRP3-related inflammatory readouts, but direct pathway perturbation and translational vascular studies are needed before causal or clinical claims can be made.

## Supporting information

S1 Fig Section-level to animal-level concordance for Oil Red O morphometry. (TIF)

S2 Fig Concordance between aortic IHC scores and RT-qPCR fold changes for caspase-1, IL-1β, MCP-1 and NLRP3. (TIF)

S3 Fig Representative flow-cytometry gating strategy for aortic macrophages and intracellular ROS. (A-D) Sequential gating of cells, singlets, CD45 + leukocytes and F4/80 + CD11b+ macrophages. (E) CD86 + M1 and CD206 + M2 gates. (F) CellROX fluorescence for intracellular ROS assessment.
(TIF)

S4 Fig Exploratory per-animal correlations between systemic cytokines and aortic mechanistic readouts. (TIF)

S5 Fig Exploratory multi-modal PCA. (TIF)

S1 Table Antibodies used in this study. (DOCX)

S1 Dataset Raw data for all quantitative analyses. (ZIP)

S1 Raw Images Uncropped original western blot images. (PDF)
