# Supplementary material for: Resmetirom attenuates atherosclerosis-associated dyslipidemia and vascular inflammation in high-fat diet-fed ApoE−/− mice
Source: PLoS One. 2026 Sep 24;21(9):e0359161. doi: 10.1371/journal.pone.0359161 (PMC13606967; doi:10.1371/journal.pone.0359161)

Figure 6A - NLRP3 (118 kDa)

Source: original uncropped Western blot image; the red box denotes the target-band region used for analysis.
Lane order after marker: NC, MC, LD, HD, NC, MC, LD, HD, NC, MC, LD, HD.
Lanes 1-4 were used in Figure 6A; lanes 5-12 were excluded from the representative panel and are marked X.
Groups: NC, normal control; MC, model control; LD, resmetirom 3 mg/kg/day; HD, resmetirom 10 mg/kg/day.
Image capture: Bio-Rad ChemiDoc MP Imaging System. Molecular-weight marker is shown at the left.

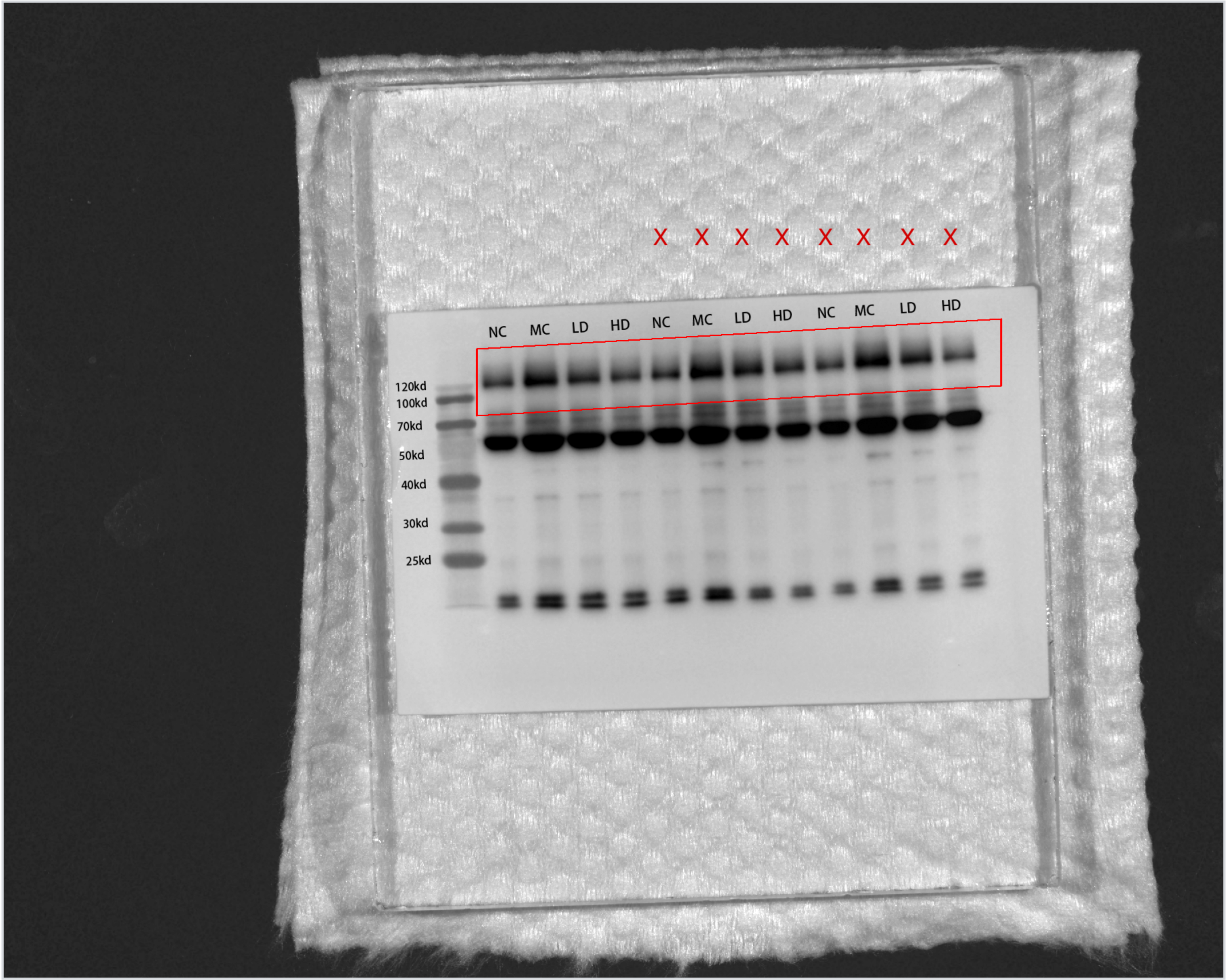

Figure 6A - caspase-1 (45 kDa)

Source: original uncropped Western blot image; the red box denotes the target-band region used for analysis.
Lane order after marker: NC, MC, LD, HD, NC, MC, LD, HD, NC, MC, LD, HD.
Lanes 1-4 were used in Figure 6A; lanes 5-12 were excluded from the representative panel and are marked X.
Groups: NC, normal control; MC, model control; LD, resmetirom 3 mg/kg/day; HD, resmetirom 10 mg/kg/day.
Image capture: Bio-Rad ChemiDoc MP Imaging System. Molecular-weight marker is shown at the left.

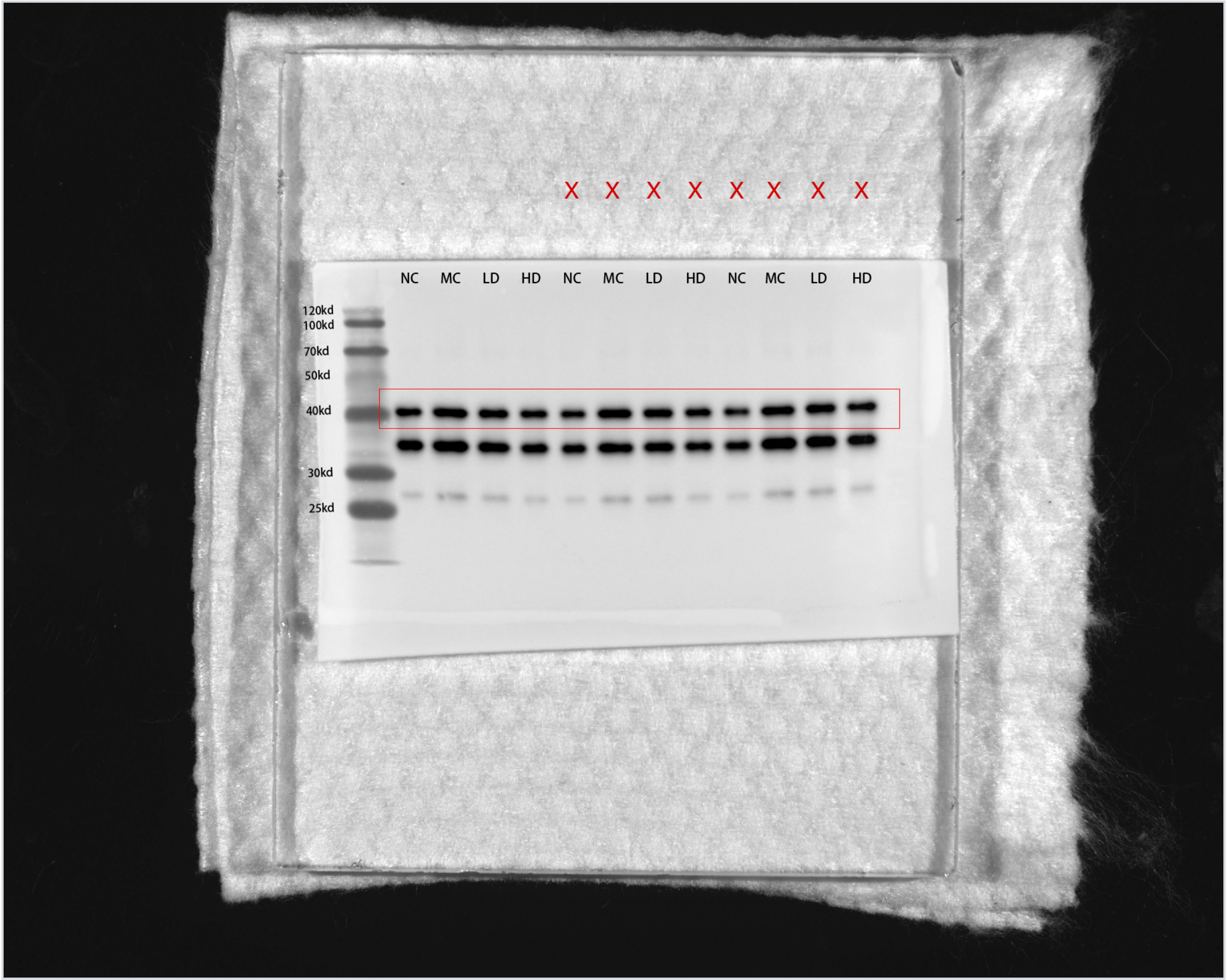

Figure 6A - IL-1β (31 kDa)

Source: original uncropped Western blot image; the red box denotes the target-band region used for analysis.
Lane order after marker: NC, MC, LD, HD, NC, MC, LD, HD, NC, MC, LD, HD.
Lanes 1-4 were used in Figure 6A; lanes 5-12 were excluded from the representative panel and are marked X.
Groups: NC, normal control; MC, model control; LD, resmetirom 3 mg/kg/day; HD, resmetirom 10 mg/kg/day.
Image capture: Bio-Rad ChemiDoc MP Imaging System. Molecular-weight marker is shown at the left.

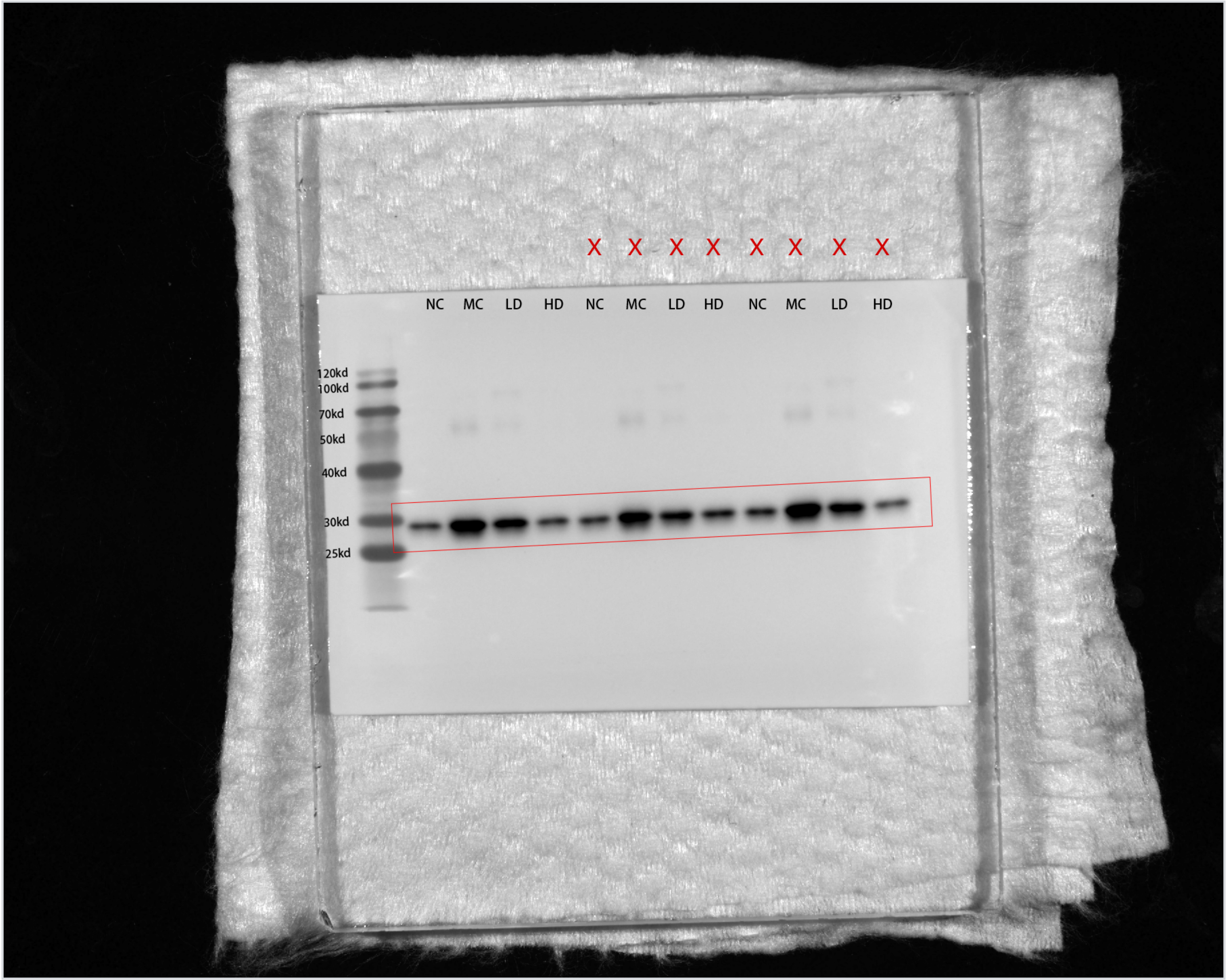

Figure 6A - MCP-1 (14 kDa)

Source: original uncropped Western blot image; the red box denotes the target-band region used for analysis.
Lane order after marker: NC, MC, LD, HD, NC, MC, LD, HD, NC, MC, LD, HD.
Lanes 1-4 were used in Figure 6A; lanes 5-12 were excluded from the representative panel and are marked X.
Groups: NC, normal control; MC, model control; LD, resmetirom 3 mg/kg/day; HD, resmetirom 10 mg/kg/day.
Image capture: Bio-Rad ChemiDoc MP Imaging System. Molecular-weight marker is shown at the left.

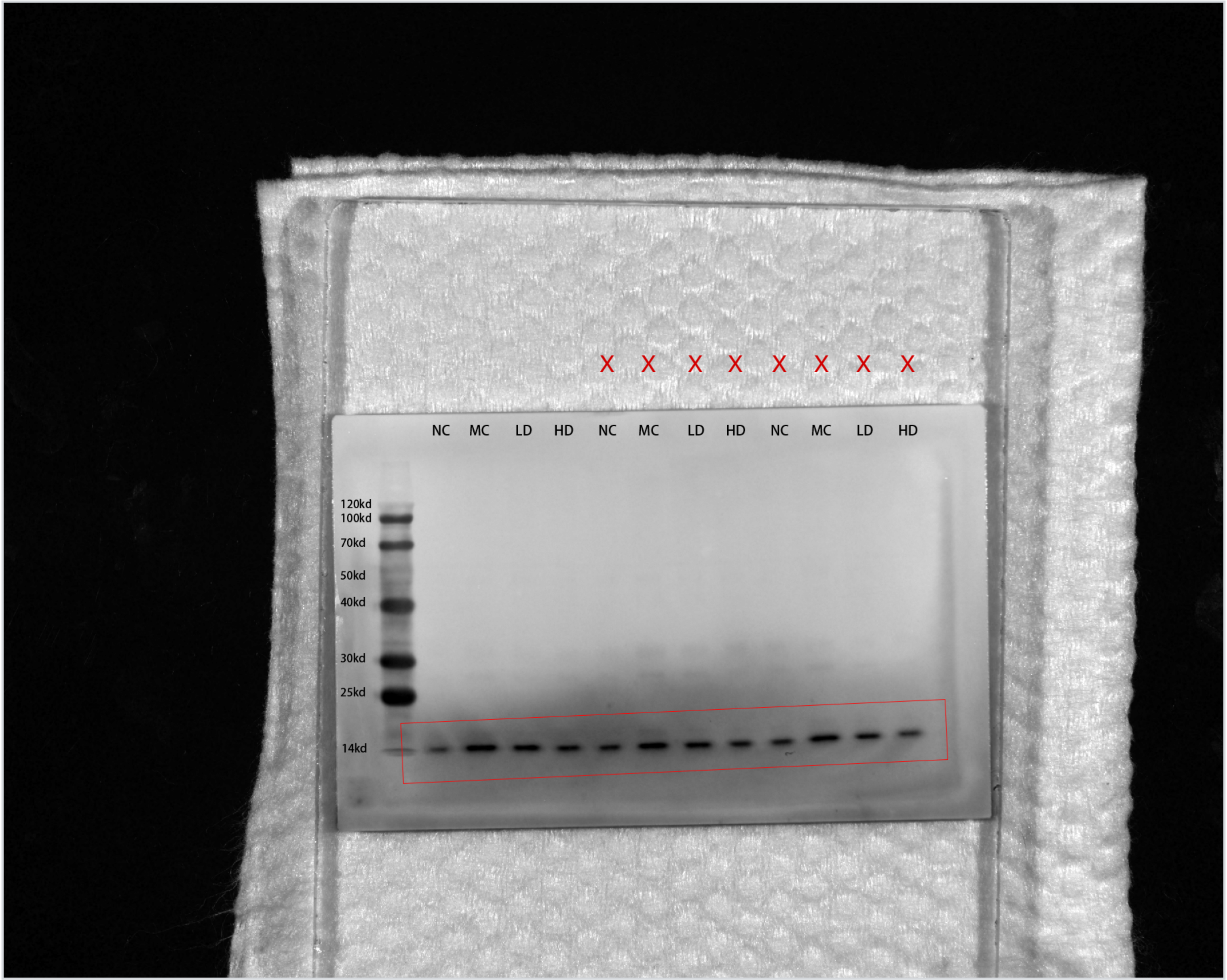

Figure 6A - phospho-p65 (Ser536) (65 kDa)

Source: original uncropped Western blot image; the red box denotes the target-band region used for analysis.
Lane order after marker: NC, MC, LD, HD, NC, MC, LD, HD, NC, MC, LD, HD.
Lanes 1-4 were used in Figure 6A; lanes 5-12 were excluded from the representative panel and are marked X.
Groups: NC, normal control; MC, model control; LD, resmetirom 3 mg/kg/day; HD, resmetirom 10 mg/kg/day.
Image capture: Bio-Rad ChemiDoc MP Imaging System. Molecular-weight marker is shown at the left.

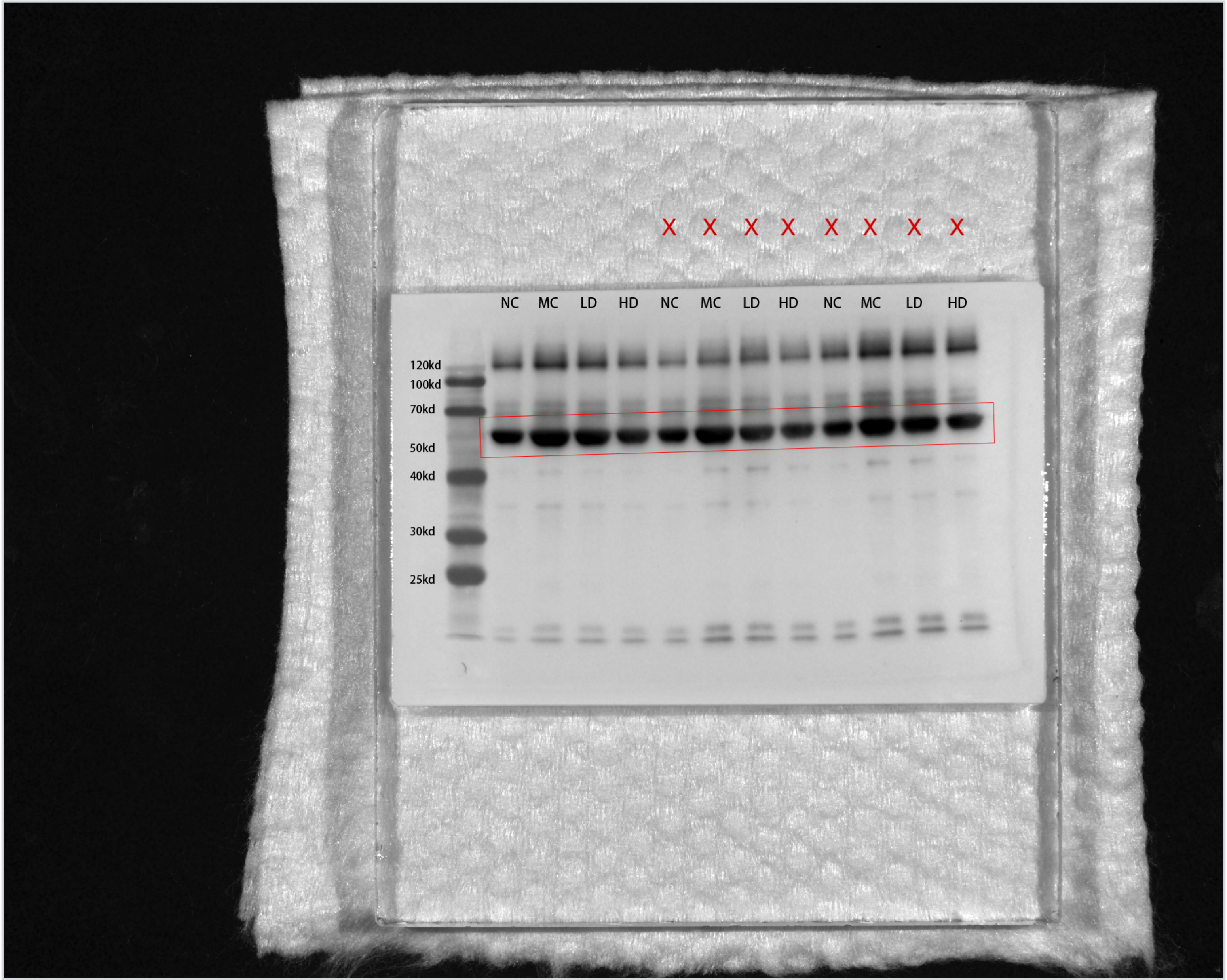

Figure 6A - total p65 (65 kDa)

Source: original uncropped Western blot image; the red box denotes the target-band region used for analysis.
Lane order after marker: NC, MC, LD, HD, NC, MC, LD, HD, NC, MC, LD, HD.
Lanes 1-4 were used in Figure 6A; lanes 5-12 were excluded from the representative panel and are marked X.
Groups: NC, normal control; MC, model control; LD, resmetirom 3 mg/kg/day; HD, resmetirom 10 mg/kg/day.
Image capture: Bio-Rad ChemiDoc MP Imaging System. Molecular-weight marker is shown at the left.

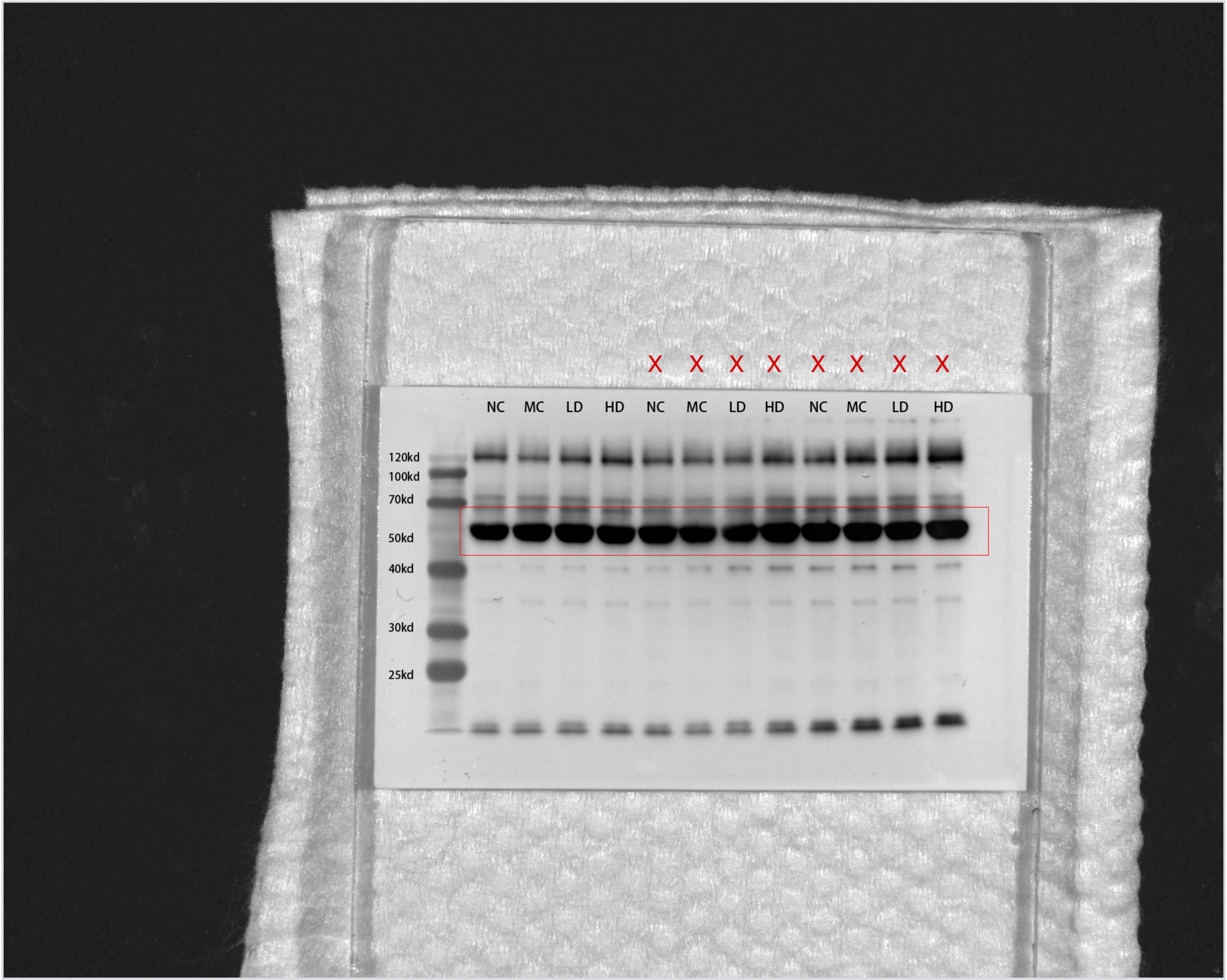

Figure 6A - IκBα (36 kDa)

Source: original uncropped Western blot image; the red box denotes the target-band region used for analysis.
Lane order after marker: NC, MC, LD, HD, NC, MC, LD, HD, NC, MC, LD, HD.
Lanes 1-4 were used in Figure 6A; lanes 5-12 were excluded from the representative panel and are marked X.
Groups: NC, normal control; MC, model control; LD, resmetirom 3 mg/kg/day; HD, resmetirom 10 mg/kg/day.
Image capture: Bio-Rad ChemiDoc MP Imaging System. Molecular-weight marker is shown at the left.

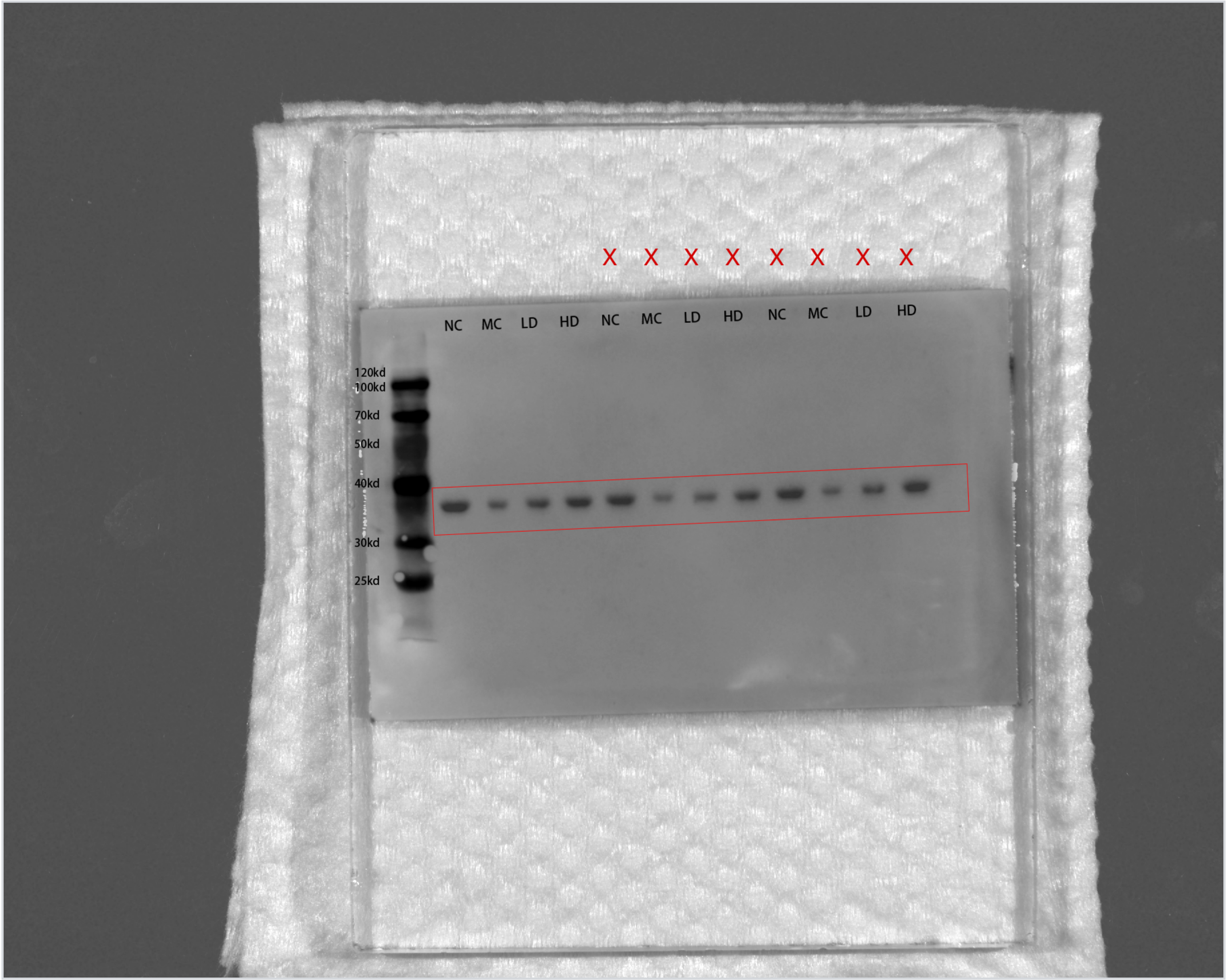

Figure 6A - GAPDH (37 kDa)

Source: original uncropped Western blot image; the red box denotes the target-band region used for analysis.
Lane order after marker: NC, MC, LD, HD, NC, MC, LD, HD, NC, MC, LD, HD.
Lanes 1-4 were used in Figure 6A; lanes 5-12 were excluded from the representative panel and are marked X.
Groups: NC, normal control; MC, model control; LD, resmetirom 3 mg/kg/day; HD, resmetirom 10 mg/kg/day.
Image capture: Bio-Rad ChemiDoc MP Imaging System. Molecular-weight marker is shown at the left.

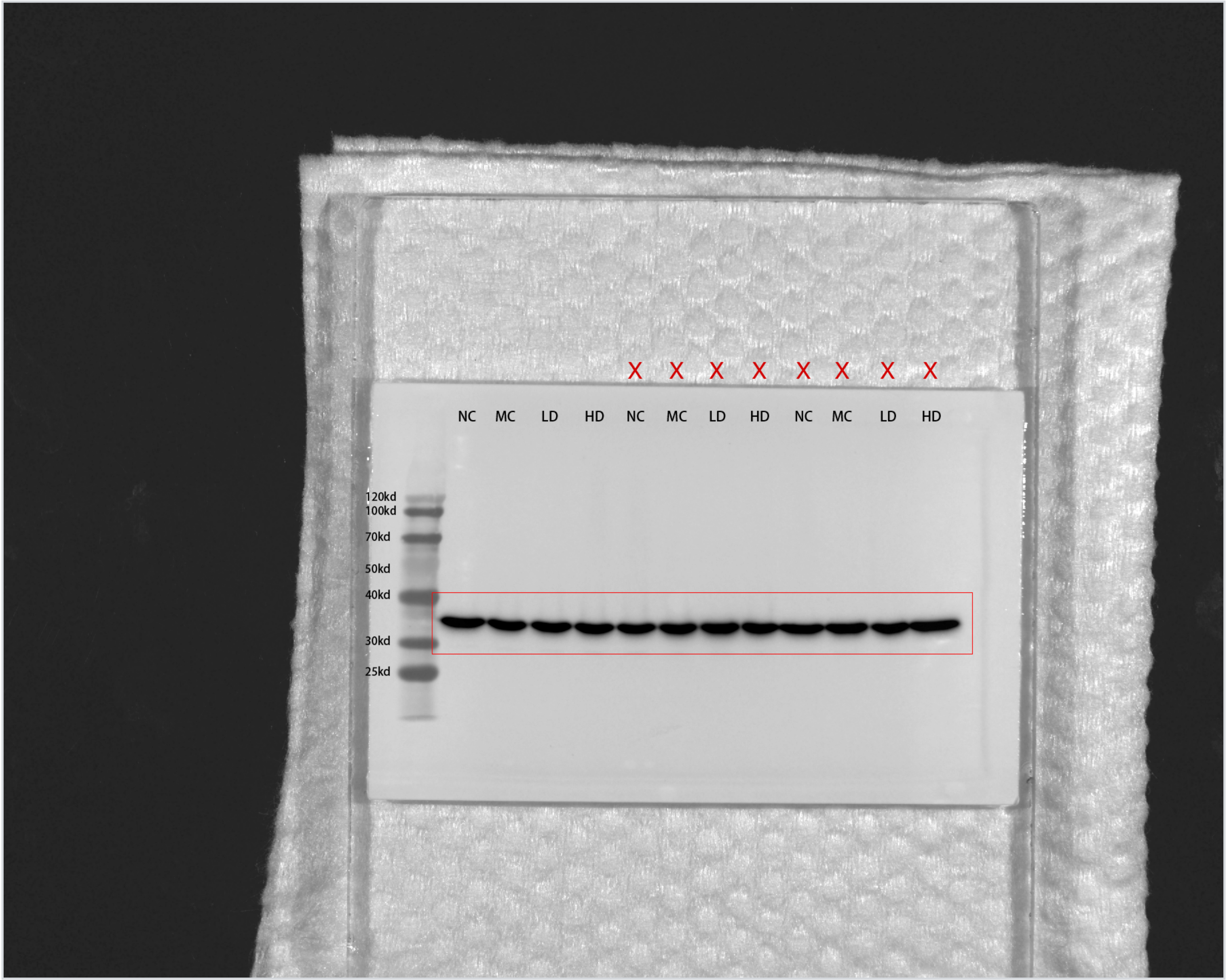

Supplement: Supplementary file 1 [file pone.0359161.s008.pdf]
