# Supplementary material for: Resmetirom attenuates atherosclerosis-associated dyslipidemia and vascular inflammation in high-fat diet-fed ApoE−/− mice
Source: PLoS One. 2026 Sep 24;21(9):e0359161. doi: 10.1371/journal.pone.0359161 (PMC13606967; doi:10.1371/journal.pone.0359161)
Supplement: Supplementary file 2 [file pone.0359161.s006.docx]

**S1 Table. Antibodies used in this study**

Antibody specifications and working conditions used for Western blotting and immunohistochemistry, together with the flow-cytometry antibody panel, are summarized below.

**Table S1A. Antibodies used for Western blotting**

| **Target** | **Antibody / host** | **Supplier** | **Catalog no.** | **Dilution** |
| --- | --- | --- | --- | --- |
| GAPDH | Rabbit anti-GAPDH | Jiangsu KeyGEN BioTECH Co., Ltd. | KGC6102-1 | 1:500 |
| Caspase-1 | Rabbit anti-Caspase-1 | BioDragon | RM8397 | 1:1,000 |
| NLRP3 | Rabbit anti-NLRP3 | Affinity Biosciences | DF7438 | 1:1,000 |
| IL-1β | Rabbit anti-IL-1β | Affinity Biosciences | AF5103 | 1:1,000 |
| MCP-1 | Rabbit anti-MCP-1 | Affinity Biosciences | AF7577 | 1:1,000 |
| Phospho-NF-κB p65 | Rabbit anti-phospho-NF-κB p65 | Affinity Biosciences | AF2006 | 1:1,000 |
| NF-κB p65 | Rabbit anti-NF-κB p65 | Affinity Biosciences | AF15006 | 1:1,000 |
| IκBα | Rabbit anti-IκBα | Affinity Biosciences | AF5002 | 1:1,000 |
| Secondary antibody | Goat anti-rabbit IgG-HRP | Jiangsu KeyGEN BioTECH Co., Ltd. | KGC6202-0.1 | 1:5,000 |

**Table S1B. Primary antibodies used for immunohistochemistry**

| **Target** | **Antibody / host** | **Supplier** | **Catalog no.** | **Dilution** |
| --- | --- | --- | --- | --- |
| IL-1β | Rabbit anti-IL-1β | Proteintech | 26048-1-AP | 1:400 |
| MCP-1 | Rabbit anti-MCP-1 (polyclonal) | Proteintech | 26161-1-AP | 1:100 |
| Caspase-1 | Rabbit anti-Caspase-1 | Proteintech | 31020-1-AP | 1:200 |
| NLRP3 | Rabbit anti-NLRP3 | Affinity Biosciences | DF7438 | 1:100 |

**Table S1C. Antibodies used for flow cytometry**

| **Marker** | **Antibody / clone** | **Supplier** | **Catalog no.** | **Fluorochrome** | **Amount used** |
| --- | --- | --- | --- | --- | --- |
| CD45 | Anti-mouse CD45 (clone 30-F11) | BioLegend | 103138 | BV510 | ≤0.5 μg/10⁶ cells/100 μL |
| CD11b | Anti-mouse/human CD11b (clone M1/70) | BioLegend | 101206 | FITC | ≤0.25 μg/10⁶ cells/100 μL |
| F4/80 | Anti-mouse F4/80 (clone BM8) | BioLegend | 123116 | APC | 0.25 μg/10⁶ cells/100 μL |
| CD86 | Anti-mouse CD86 (clone GL-1) | BioLegend | 105008 | PE | 0.25 μg/10⁶ cells/100 μL |
| CD206 | Anti-mouse CD206/MMR (clone C068C2) | BioLegend | 141720 | PE-Cy7 | ≤0.25 μg/10⁶ cells/100 μL |

Abbreviations: APC, allophycocyanin; FITC, fluorescein isothiocyanate; HRP, horseradish peroxidase; IHC, immunohistochemistry; PE, phycoerythrin.
